# The Alleviation of Heat Damage to Photosystem II and Enzymatic Antioxidants by Exogenous Spermidine in Tall Fescue

**DOI:** 10.3389/fpls.2017.01747

**Published:** 2017-10-12

**Authors:** Liang Zhang, Tao Hu, Erick Amombo, Guangyang Wang, Yan Xie, Jinmin Fu

**Affiliations:** ^1^Key Laboratory of Plant Germplasm Enhancement and Specialty Agriculture, Wuhan Botanical Garden, Chinese Academy of Sciences, Wuhan, China; ^2^Graduate University of Chinese Academy of Sciences, Beijing, China; ^3^School of Resources and Environmental Engineering, Ludong University, Yantai, China

**Keywords:** spermidine, tall fescue, heat stress, antioxidant enzymes, photosystem II, gene expression

## Abstract

Tall fescue (*Festuca arundinacea* Schreb) is a typical cool-season grass that is widely used in turf and pasture. However, high temperature as an abiotic stress seriously affects its utilization. The objective of this study was to explore the effect of spermidine (Spd) on heat stress response of tall fescue. The samples were exposed to 22°C (normal condition) or 44°C (heat stress) for 4 h. The results showed that exogenous Spd partially improved the quality of tall fescue leaves under normal temperature conditions. Nevertheless, after heat stress treatment, exogenous Spd significantly decreased the electrolyte leakage of tall fescue leaves. Spd also profoundly reduced the H_2_O_2_ and O_2_^⋅-^ content and increased antioxidant enzymes activities. In addition, PAs can also regulate antioxidant enzymes activities including SOD, POD, and APX which could help to scavenge ROS. Moreover, application of Spd could also remarkably increase the chlorophyll content and had a positive effect on the chlorophyll α fluorescence transients under high temperature. The Spd reagent enhanced the performance of photosystem II (PSII) as observed by the JIP-test. Under heat stress, the Spd profoundly improved the partial potentials at the steps of energy bifurcations (PI_ABS_ and PI_total_) and the quantum yields and efficiencies (φP_0_, δR_0_, φR_0_, and γRC). Exogenous Spd could also reduce the specific energy fluxes per Q_A_^-^ reducing PSII reaction center (RC) (TP_0_/RC and ET_0_/RC). Additionally, exogenous Spd improved the expression level of *psbA* and *psbB*, which encoded the proteins of PSII core reaction center complex. We infer that PAs can stabilize the structure of nucleic acids and protect RNA from the degradation of ribonuclease. In brief, our study indicates that exogenous Spd enhances the heat tolerance of tall fescue by maintaining cell membrane stability, increasing antioxidant enzymes activities, improving PSII, and relevant gene expression.

## Introduction

Tall fescue (*Festuca arundinacea* Schreb) is a major cool-season grass that is widely used for turf, on the sports field, and as a forage grass with an optimal growth temperature of 16–24°C (Emmons, 2007). However, it is sensitive to heat stress which affects tall fescue turf quality and utilization. When the temperature exceeds the optimal range, heat stress could lead to the photosynthesis inhibition, cell membrane damage, senescence, severe obstruction in growth, development, and even death (Xu et al., 2006; Mostofa et al., 2014). Therefore, the high temperature is the key limiting factor for tall fescue utilization. It is crucial to explore a convenient method to reduce the damage of tall fescue by heat stress.

Heat stress brings great challenge to the utilization of cool-season turfgrass worldwide. It results in the loss of balance between the scavenging and producing of reactive oxygen species (ROS) (Smirnoff, 1998). ROS can be produced constantly in the process of plant growth and development which includes hydrogen peroxide (H_2_O_2_), singlet oxygen (^1^O_2_), hydroxyl radical (OH^⋅^), and superoxide anion (O_2_^⋅-^) (Mostofa et al., 2014). Under normal circumstances, the balance of generation and ROS scavenging is harmless to plants. When the botanical species are under environmental stress including heat, on one hand, the accumulation of ROS can lead to injury to the cell membrane via increasing electrolyte leakage (EL) (Liu and Huang, 2000). On the other hand, heat stress could also decrease the activities of antioxidants causing injury to plants (Monk et al., 1989). Overproduction of ROS also has negative influences on biomacromolecule containing membrane lipids, proteins, nucleic acids, and chlorophyll which are necessary for growth and development to plants (Blokhina et al., 2003). The evolution of higher plants has developed the ROS-scavenging system to defend against oxidative stress (Mittler et al., 2004). Enzymatic antioxidants include superoxide dismutase (SOD), catalase (CAT), peroxidase (POD), and ascorbate peroxidase (APX) which can scavenge H_2_O_2_, O_2_^⋅-^, and oxy-intermediates (Apel and Hirt, 2004; Lee et al., 2007). The SOD is regarded as the first line of defense against ROS and catalyzes O_2_^⋅-^ to H_2_O_2_ and oxygen (Sigaud-Kutner et al., 2002), while H_2_O_2_ can be further removed by POD and APX through dismutation or ascorbateglutathione cycle (Mostofa et al., 2014; Liu et al., 2016).

Photosynthesis involves a series of complex metabolic reactions which are not only vital for biological survival but also forms a critical carbon-oxygen balance on earth (Berry and Downton, 1982). The structural and functional photosynthetic machinery is sensitive and vulnerable to severe or mild heat stress (Essemine et al., 2011). Photosystem II (PSII) as the core portion of the photosynthetic process whose components are susceptible to high-temperature stress and are seriously damaged (Hideg et al., 2002). PSII located in the thylakoid membranes of oxygenic photosynthetic organisms is a membrane protein complex with multi-subunit that catalyzes a series of electron transfer reactions (Umena et al., 2011). Basically, the PSII catalyzes the unique reactions resulting in the splitting of water and the production of dioxygen and reducing equivalents (Barber, 1998). The PS II is composed of two different parts of structure and function. One as the reaction center (RC) of PS II is constituted by a D1-D2 heterodimer binding capture complex named CP43 and CP47. The other part is the pigment protein complex which binds plenty of Chl a, Chl b, and lutein (Barber, 1998; Pfannschmidt, 2003; Pospíšil, 2012). Thereinto, D1 protein is the most important subunit which can provide a position for cofactors to bind, maintain the structure of PS II reaction center, and have a close connection with the separation and transmission of the original charge (Kruse et al., 1997).

It has been previously reported that many low molecular compounds have an essential role in plants to respond to abiotic stress. Bartwal et al. (2013) reported that brassinosteroids could enhance the tolerance of chilling, heat, salt, and drought in rice, tomato, beet, and wheat by maintaining membrane stability and modulating the expression of relevant genes. Tan et al. (2011) study showed that CaCl_2_ treatment has a positive effect on improving heat tolerance of tobacco by elevating net photosynthetic rate, thermostability of reaction center of PSII, antioxidative enzymes activity, and HSP70 level. Salicylic Acid has been found to exert some positive effects on the improvement of malting barley resistances to heavy metals by increasing the activities of SOD and CAT (Song et al., 2014). Expect above, polyamines (PAs), including putrescine (Put), spermidine (Spd), and spermine (Spm), is one of the vital compounds. The PAs play crucial roles in various abiotic stresses, including salt, drought, high temperature, wounding, ozone, flooding, heavy metals, acid, and oxidative stresses (Shi and Chan, 2014). PAs are a type of ubiquitous nitrogenous compounds containing two or more amines and exist in almost all organisms (Alcázar et al., 2010). They are widely involved in the regulation of growth and development in plants, such as morphogenesis, root elongation, pollen viability, leaf senescence, fruit ripening, programmed cell death, DNA synthesis, gene transcription, protein translation, and chromatin organization (Shi and Chan, 2014). Additionally, PAs are also considered as vital secondary messengers in the signaling pathway (Kusano et al., 2008). They could maintain membrane stability by their interaction with phospholipids as well as scavenge ROS (Roberts et al., 1986; Besford et al., 1993). PAs will largely accumulate under abiotic stresses including high temperature (Todorova et al., 2007; Goyal and Asthir, 2010).

Exogenous application low molecular compound is regarded as one of the efficient methods to alleviate environmental stresses of plants. There is tremendous progress concerning exogenous PAs response to abiotic stresses in rice, tomato, and cucumber (Tian et al., 2012; Mostofa et al., 2014; Hu L. et al., 2016). Most of them had a close association with enhanced levels of antioxidant capacity. However, studies on the effect of Spd on PSII and the expression of relevant genes in tall fescue under heat stress have still been obscure. Therefore, the objectives of this study were to explore the influence of Spd on chlorophyll α fluorescence, antioxidant enzyme activity, and photosynthetic gene transcription level in tall fescue under high temperature to enrich the information in our attempts to comprehend turf breeding and management.

## Materials and Methods

### Plant Materials and Growth Conditions

This research was conducted at Wuhan Botanical Garden, Chinese Academy of Sciences, Wuhan, China in 2016. The plant used in this study was PI234881 seeded in plastic pots (13 cm in diameter and 15 cm deep) with matrix (brown coal soil and sand = 1:1). Plants were maintained in the greenhouse after germination with day/night temperature 22/18°C (± 2°C), humidity 80% and illumination 14 h (with light intensity of 300 μmol photons m^-2^ s^-1^) for 50 days. The seedlings were watered daily and fertilized once a week with 100 mL of half-strength Hoagland’s solution (Hoagland and Arnon, 1950).

### Reagent Treatments

The reagent treatments were performed using a vacuum infiltration to allow efficient transportion of reagents into the leaves (Filippou et al., 2012) as described by Carolina et al. and Luisa et al. with little modification (Ederli et al., 2006; Attallah et al., 2007). The collection was done by snipping third fully expanded leaves from petiole which were subsequently immersed in half-strength Hoagland’s solution with various pharmacological reagents in 15 cm length containers. Later, the leaves were infiltrated with a desiccator under dark vacuum condition for 15 min where they are maintained for 4 h to enable fully recovering before heat stress treatment.

### Heat Stress Treatment

For the heat stress treatment, the leaf petioles were immersed in a 1-cm deep half-strength Hoagland’s solution kept in falcon tubes. Subsequently, the tubes were transferred into two growth chambers with temperature regimes of 22°C (CK and S) or 44°C (H and HS). The light intensity was set at 450 μmol photons m^-2^ s^-1^ at 80% humidity. Each treatment was repeated at least three times.

### Evaluation of the Optimum Spd Concentration

**FIGURE 1:**
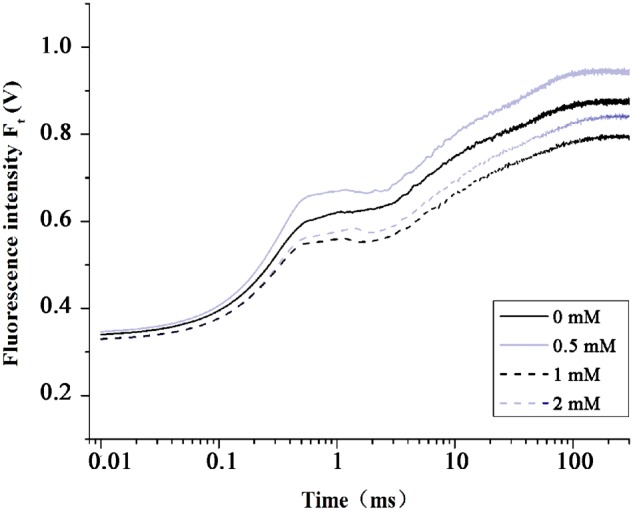
Evaluation of the optimum Spd concentration by OJIP curves. The OJIP fluorescence transients in tall fescue leaves with 0, 0.5, 1, and 2 mM Spd at high temperature (44°C) for 4 h.

To determine the adequate effective Spd concentration for alleviating heat stress, we performed a preliminary experiment by applying different concentration Spd. The concentration of Spd (0, 0.5, 1, and 2 mM) were chosen preliminarily according to the Mostofa experiment on rice (Mostofa et al., 2014). Subsequently, we selected the optimum concentration (0.5 mM) by comparing the fluorescence transients after heat stress 4 h (**Figure 1**). **Figure 1** shows the differential changes in chlorophyll fluorescence transients after treatment with different concentration of Spd under heat stress. A 0.5 mM of Spd had the positive impact on photosynthesis by improving F_J_, F_I_, and F_P_.

### Chlorophyll Content

The leaf chlorophyll content was measured based on the method described by Hiscox and Israelstam (1979). Briefly, fresh leaves (0.1 g) were immersed in a 10-mL dimethylsulfoxide tube, then the absorbance of the samples at 645 and 663 nm was measured by spectrophotometer (UV-2600, UNICO, Shanghai) after 72 h dark treatment. The chlorophyll content was calculated by the following formula:

Chl a (mg/g FW) = (12.72 ^∗^ OD663 – 2.59 ^∗^ OD645) ^∗^ 0.3

Chl b (mg/g FW) = (22.88 ^∗^ OD645 – 4.67 ^∗^ OD663) ^∗^ 0.3

Chl total (mg/g FW) = (20.2 ^∗^ OD645 + 8.02 ^∗^ OD663) ^∗^ 0.3.

Where OD645 and OD663 are the absorbance of the extract solution at 645 and 663 nm, respectively, and FW is the fresh weight of the leaf.

### Electrolyte Leakage (EL)

To quantify the EL, 0.1 g of treated leaves were washed three times with deionized water. The leaves were cut into 0.5 cm long debris and put into test tubes filled with 15 mL deionized water. The tubes were shaken for 24 h at 25°C and the initial conductivity (C_i_) was measured by a conductivity meter (JENCO-3173, Jenco Instruments, Inc., San Diego, CA, United States). Subsequently, the leaves were autoclaved at 121°C for 30 min to release the electrolytes of the tissue completely. The final conductivity (C_max_) was measured after the solution had been cooled to room temperature. The relative EL was calculated with the formula:

EL (%) = (C_i_/C_max_) ^∗^ 100%.

### Crude Enzyme Extraction

For enzyme extracts, a 0.2 g of leaves powder with liquid nitrogen was immersed in 4 mL phosphate buffer (150 mM, pH 7.0) precooled at 4°C homogenized with 0.2 M Na_2_HPO_4_ and 0.2 M NaH_2_PO_4_. Then, the homogenate was centrifuged at 15,000 × *g* at 4°C for 30 min. Finally, the supernatant was collected and stored at 4°C to determine enzyme activities.

### Antioxidant Enzyme Activity

For the SOD activity assay, a 0.1 mL enzyme extract was added into 2.9 mL solution plus 50 mM phosphate buffer (pH 7.8), 1.125 mM nitro blue tetrazolium (NBT), 60 μM riboflavin, 195 mM methionine and 3 μM ethylene diamine tetraacetic acid (EDTA). Then, the solution was incubated under 4000 lx irradiance for 30 min. The change of absorbance at 560 nm was recorded with 3 mL of solution without enzyme extract as the control. One unit of SOD activity was defined as the inhibition of NBT reduction by 50%.

The POD activity was measured based on the method described by Fan et al. (2014). In brief, a 50 μL enzyme extract was added into 2.95 mL solution containing 0.075% H_2_O_2_, 0.1 M sodium acetate-acetic buffer (pH 5.0), 0.25 mL guaiacol (dissolved in 50% ethanol solution). Then we recorded the absorbance changes at 460 nm per minute for 3 min. One unit POD activity is defined as the increase in absorbance per minute.

The APX activity was measured using Plant APX Elisa Kit (Huijia Biotechnology Institute, Xiamen, China).

### H_2_O_2_ and O_2_^•-^ Content

The H_2_O_2_ content was determined using a H_2_O_2_ Assay Kit (Nanjing Jiancheng Bioengineering Institute, China).

The O_2_^•-^ content was measured using Plant SOA Elisa Kit (Huijia Biotechnology Institute, Xiamen, China).

### Chlorophyll (Chl) α Fluorescence Transient

Chlα fluorescence transients were recorded by pulse-amplitude modulation (PAM) fluorometer (PAM 2500, Heinz Walz GmbH). After 30 min of adaption in the darkness, leaves were triggered with the red light of 3000 μmol photons m^-2^ s^-1^ to attain OJIP transients which were measured and digitized between 10 and 320 ms. The data of OJIP transients analyzed method was initially reported by Strasser et al. (2004). In the present assay, the data of OJIP transients were analyzed by using JIP-test as reported by Chen et al. (2014). The JIP-test is used for analysis parameters of OJIP transient, which is based on the energy fluxes in the biofilm. These parameters digitize photosynthesis to further study of the photosystem.

### Quantitative RT-PCR Analysis

The levels of gene expression were analyzed by approximately 0.1 g crushed leaves. Total RNA was extracted and purified by Trizol-reagent (Invitrogen, Carlsbad, CA, United States) according to the instruction. About 0.2 μg RNA was used for synthesizing the first-strand cDNA using M-MLV reverse transcriptase (Promega, Madison, WI, United States) with an oligo (dT) primer. Then we examined the quality of cDNA by gel electrophoresis and maintained it at -80°C for qRT-PCR analysis. Specific primers (**Table 1**) were designed for analyzing gene expression, fluorescent dye SYBR Green (Toyobo, Osaka, Japan) was applied in the detection system. Real-time PCR reaction was performed by the real-time PCR Master Mix (Toyobo) according to the manual. The TUB gene was used as an internal control. The method used to determine the relative quantity of the target gene expression was proposed by Chen et al. (2009).

**Table 1:** Primer sequences and information used for reserved transcription real-time PCR (RT-PCR) analyses.

Gene	Encoded polypeptide		Primers sequences (5′–3′)	Size (bp)	Gene ID
*psb A*	D1 protein	F	GTATTTATTATCGCCTTCATCG	284	7095419
		R	AGGACGCATACCCAAACG		
*psb B*	CP47	F	TAGGCGTAACGGTGGA	254	7095420
		R	AACATCTCGGAACAAGG		
*psb C*	CP43	F	TAATACGGCTTATCCGAGTGAGTTT	288	7095484
		R	TCTTGCCAAGGTTGTATGTCTTT		

*F and R represent forward and reverse, respectively.*

### Statistical Analysis

In the experiment, all values were shown as mean ± (Standard Error) SE with at least triplication. One-way analysis of variance (ANOVA) and Duncan’s multiple range test were conducted using SPSS (version 20.0, SPSS Institute, Chicago, IL, United States) statistical software and 5% level of probability was used to test the significant effects of treatments. The graphs were produced by Origin 8.0 (Origin Lab, Inc., Hampton, MA, United States) and Excel 2010 for Windows.

## Results

### Electrolyte Leakage

**FIGURE 2:**
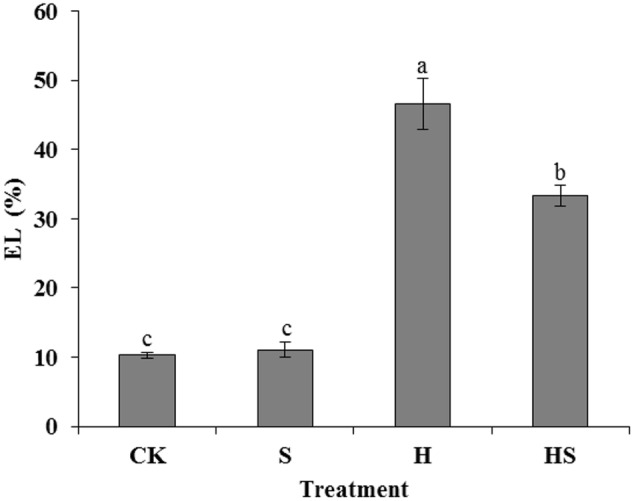
Effects of EL in tall fescue leaves after 0.5 mM Spd treatment under heat stress. CK was normal temperature of 22°C. H was high temperature where tall fescue leaves were treated at 44°C. S was treated with Spd at normal temperature. HS was treated with Spd at high temperature. Values are given as means ± SE of three independent experiments. Different letters indicate statistical difference significance at *P* < 0.05 among the treatments by Duncan’s multiple range test.

The EL alteration was measured to investigate the role of exogenous Spd in maintaining cell membrane stability of tall fescue under heat stress. The result showed that EL values in the leaves of control and Spd treatment regime had no significant difference. However, under heat stress, the EL value increased five folds, compared to the control. The application of Spd to stressed leaves significantly reduced EL by 28.64% compared to heat stress treatment (**Figure 2**).

### Chlorophyll Content

**FIGURE 3:**
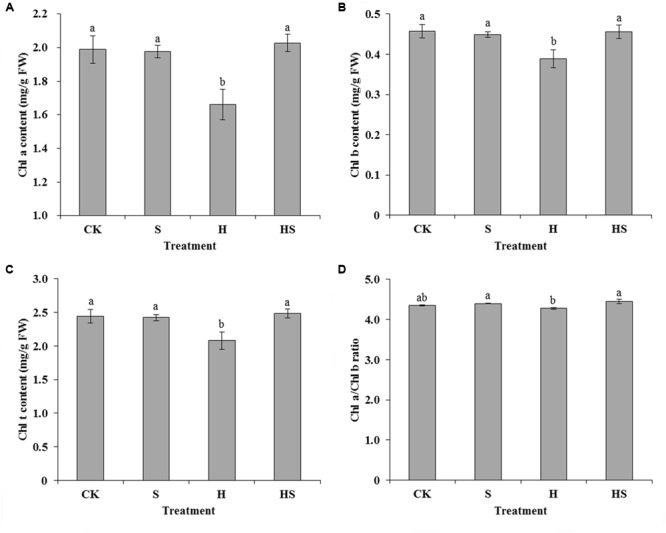
Effects of chlorophyll content in tall fescue leaves after 0.5 mM Spd treatment under heat stress. **(A)** chlorophyll a content; **(B)** chlorophyll b content; **(C)** total chlorophyll content; **(D)** ratio of chlorophyll a to b. CK was normal temperature of 22°C. H was high temperature where tall fescue leaves were treated at 44°C. S was treated with Spd at normal temperature. HS was treated with Spd at high temperature. Values are given as means ± SE of three independent experiments. Different letters indicate statistical difference significance at *P* < 0.05 among the treatments by Duncan’s multiple range test.

When plants were exposed to various abiotic stresses, the leaves would exhibit chlorosis and a significant change in the chlorophyll content. Therefore, chlorophyll content is usually taken as an indicator that reflected plants resistance to stress. Under the control condition, there was no significant difference in the Chl a, Chl b, total chlorophyll content, and the ratio of Chl a to Chl b (**Figure 3**). Conversely, after heat stress treatment, Chl a, Chl b, and the total chlorophyll content notably decreased by 16.47, 14.95, and 14.96%, respectively. Interestingly, Spd application under heat stress could notably enhance the Chl a, Chl b, total chlorophyll content, and the ratio of Chl a to Chl b by 22.04, 17.32, 19.40, and 4.06%, respectively.

### Antioxidant Enzyme Activities

**FIGURE 4:**
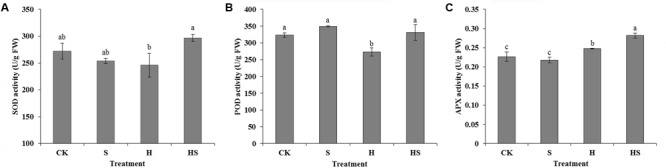
Effects of antioxidant enzyme activities in tall fescue leaves after 0.5 mM Spd treatment under heat stress. **(A)** activity of superoxide dismutase (SOD); **(B)** activity of peroxidase (POD); **(C)** activity of ascorbate peroxidase (APX). CK was normal temperature of 22°C. H was high temperature where tall fescue leaves were treated at 44°C. S was treated with Spd at normal temperature. HS was treated with Spd at high temperature. Values are given as means ± SE of three independent experiments. Different letters indicate statistical difference significance at *P* < 0.05 among the treatments by Duncan’s multiple range test.

To investigate the effect of Spd on the antioxidant enzymes, several antioxidant enzymes activities were assayed (**Figure 4**), which included SOD, POD, and APX activities. Under normal temperature, SOD activity had no change after applied Spd. However, after heat stress, the activity of SOD decreased by 9.59%. Under the heat stress, the activity of SOD after Spd treatment increased significantly by 20.67%. Heat stress damaged the activity of POD which decreasing by 15.74% compared to control. After treatment with Spd, POD activity increased significantly by 21.51%, returned to normal levels. The activity of APX was also remarkably elevated 13.52% that treated by Spd under high temperature.

#### H_2_O_2_ and O_2_^⋅-^ Contents

**FIGURE 5:**
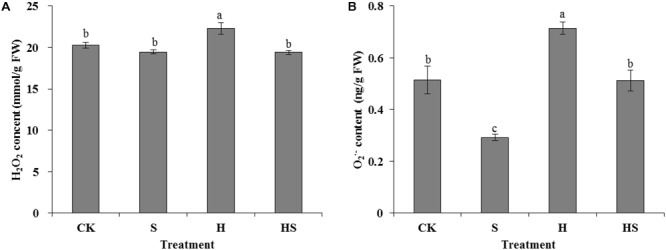
Effects of H_2_O_2_ and O_2_**^∙-^** content in tall fescue leaves after 0.5 mM Spd treatment under heat stress. **(A)** H_2_O_2_ content; **(B)** O_2_**^∙-^** content. CK was normal temperature of 22°C. H was high temperature where tall fescue leaves were treated at 44°C. S was treated with Spd at normal temperature. HS was treated with Spd at high temperature. Values are given as means ± SE of three independent experiments. Different letters indicate statistical difference significance at *P* < 0.05 among the treatments by Duncan’s multiple range test.

There was not a notable difference of H_2_O_2_ content in leaves under normal temperature whether application Spd or not (**Figure 5**). However, after heat treatment, the H_2_O_2_ content significantly increased about 10% compared to the control. Interestingly, the Spd treatment decreased the H_2_O_2_ content remarkably to normal condition. Similarly, under normal temperature, the content of O_2_^⋅-^ decreased sharply by 43.3% after applied Spd treatment, then it rose again in the leaves after heat stress treatment. However, the Spd treatment reduced the O_2_^⋅-^ contents to a normal level.

#### The OJIP Fluorescence Transient and JIP-Test

**FIGURE 6:**
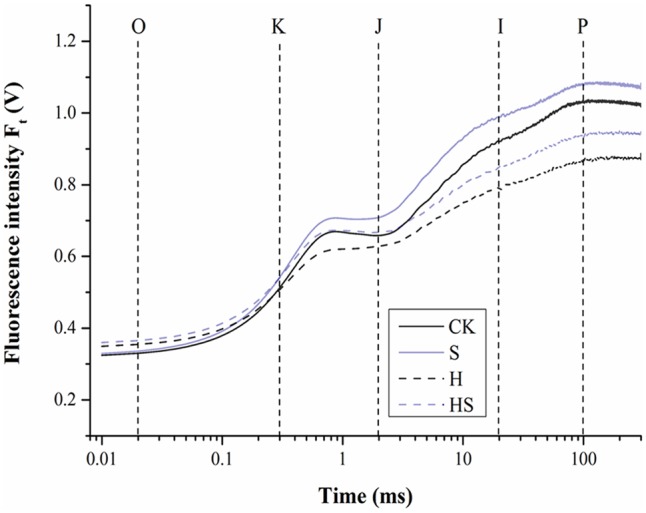
The effect of chlorophyll fluorescence transients (OJIP curve) in tall fescue leaves after 0.5 mM Spd treatment under heat stress. CK was normal temperature of 22°C. H was high temperature where tall fescue leaves were treated at 44°C. S was treated with Spd at normal temperature. HS was treated with Spd at high temperature. Values are given as means ± SE of three independent experiments. Different letters indicate statistical difference significance at *P* < 0.05 among the treatments by Duncan’s multiple range test.

On one hand, under normal temperature, Spd application increased the OJIP curve compared to non-Spd treated regime (**Figure 6**). On the other hand, heat stress treatment made the OJIP curve decline dramatically after 4 h, which was partially ameliorated by Spd treatment.

To further study the effect of Spd on the photosynthetic system under heat stress, we employed the JIP-test to analyze the parameters of OJIP transient curves. We extracted F_0_, F_K_, F_J_, F_I_, F_P_, and M_0_ as basic parameters as displayed in **Table 2**. As shown, there was no difference in the F_K_ among all treatments. However, heat stress treatment profoundly reduced the F_J_, F_I_, F_M_, while increased the F_0_, M_0_. Meanwhile, the parameters had the tendency return normal values in Spd treatment under high temperature. The leaves treated with high temperature without Spd had the highest F_0_, M_0_, and the lowest other parameters.

**Table 2:** Basic photosynthetic parameters extracted from the OJIP transient curves.

Treatment	*F*_0_	*F*_K_	*F*_J_	*F*_I_	*F*_M_	*M*_0_
CK	0.25 ± 0.01b	0.57 ± 0.01a	0.67 ± 0.02b	0.97 ± 0.01a	1.05 ± 0.01a	1.59 ± 0.08b
S	0.26 ± 0.02b	0.59 ± 0.01a	0.72 ± 0.02a	1.00 ± 0.02a	1.08 ± 0.02a	1.61 ± 0.03b
H	0.31 ± 0.01a	0.57 ± 0.01a	0.61 ± 0.01c	0.82 ± 0.01c	0.87 ± 0.01c	1.83 ± 0.05a
HS	0.28 ± 0.01ab	0.58 ± 0.01a	0.71 ± 0.01ab	0.88 ± 0.01b	0.98 ± 0.01b	1.75 ± 0.02ab

*CK was normal temperature of 22°C. H was high temperature where tall fescue leaves were treated at 44°C. S was treated with Spd at normal temperature. HS was treated with Spd at high temperature. Values are given as means ± SE of three independent experiments. Different letters indicate statistical difference significance at *P* < 0.05 among the treatments by Duncan’s multiple range test.*

**FIGURE 7:**
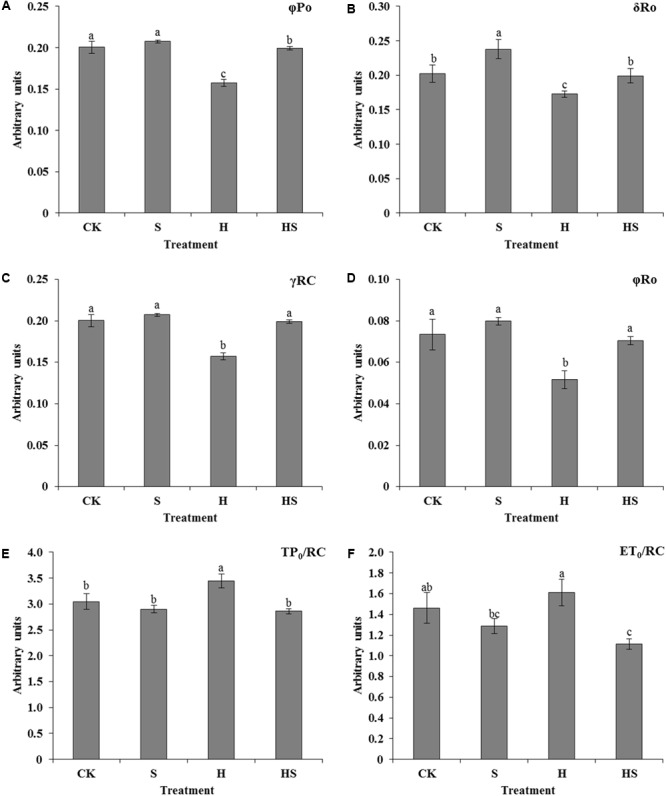
Effects of photosynthetic parameters deduced from the JIP-test analysis of fluorescence transients. **(A–D)** Alteration of quantum yields and efficiencies/probabilities; **(E,F)** alteration of energy fluxes per active PSII reaction center (RC). CK was normal temperature of 22°C. H was high temperature where tall fescue leaves were treated at 44°C. S was treated with Spd at normal temperature. HS was treated with Spd at high temperature. Values are given as means ± SE of three independent experiments. Different letters indicate statistical difference significance at *P* < 0.05 among the treatments by Duncan’s multiple range test.

All of the above basic parameters were analyzed by the JIP-test to deduce further the structural and functional parameters to quantify the photosynthesis of tall fescue leaves. There was almost no significant difference except for the value of δR_0_ in control and Spd treatment on the condition of normal temperature. In terms of quantum yields and efficiencies or probabilities, heat stress decreased the values of φP_0_ (maximum quantum yield), δR_0_ (efficiency with which an electron from Q_B_ is transferred until PSI acceptors), γRC (probability that PSII Chl molecule functions as RC), and φR_0_ (quantum yield for reduction of end electron acceptors at the PSI acceptor side) compared to normal condition. On the other hand, exogenous Spd notably enhanced the values of the φP_0_, δR_0_, γRC, and φR_0_ (**Figures 7A–D**).

Meanwhile, several parameters which were also analyzed to specify energy fluxes were displayed in **Figures 7E,F**. In the absence of heat stress, there were no obvious effects on the TP_0_/RC (trapped excitation flux per RC), and ET_0_/RC (electron transport flux per RC) for CK and Spd treatments. However, the values of TP_0_/RC and ET_0_/RC were higher under high temperature, while these values reduced after Spd application.

**FIGURE 8:**
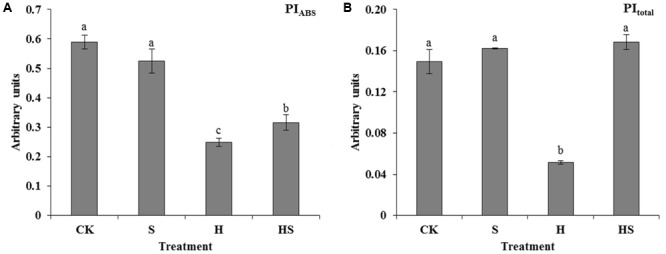
Alterations of performance index (PI) as induced by JIP-test analysis of fluorescence transients. **(A)** Alteration of PI for energy conservation from exciton to the reduction of intersystem electron (PI_ABS_); **(B)** Alteration of PI for energy conservation from exciton to the reduction of PSI end acceptors (PI_total_). CK was normal temperature of 22°C. H was high temperature where tall fescue leaves were treated at 44°C. S was treated with Spd at normal temperature. HS was treated with Spd at high temperature. Values are given as means ± SE of three independent experiments. Different letters indicate statistical difference significance at *P* < 0.05 among the treatments by Duncan’s multiple range test.

Performance index (PI) including PI_total_ and PI_ABS_ are important indices to describe the overall activity of PSII. It was shown in the **Figure 8** that PI was much higher in those under heat stress. After exogenous Spd treatment, PI_ABS_ became conspicuously higher than the leaves with heat treatment. In addition, the PI_total_ increased by approximately two folds compared with the high-temperature treatment.

### Relevant Gene Expression to Photosynthetic System

**FIGURE 9:**
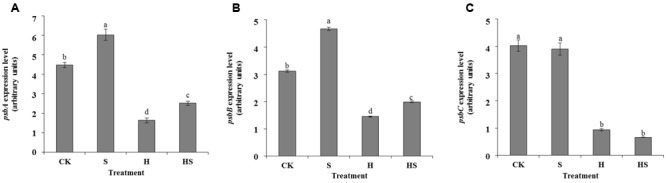
Effects of gene transcription in tall fescue leaves after 0.5 mM Spd treatment under heat stress. **(A)** *psbA* expression level; **(B)** *psbB* expression level; **(C)** *psbC* expression level. CK was normal temperature of 22°C. H was high temperature where tall fescue leaves were treated at 44°C. S was treated with Spd at normal temperature. HS was treated with Spd at high temperature. Values are given as means ± SE of three independent experiments. Different letters indicate statistical difference significance at *P* < 0.05 among the treatments by Duncan’s multiple range test.

Three genes, *psbA, psbB*, and *psbC*, encoding D1 protein, CP47, and CP43 involved in the photosynthetic system were measured with expression levels to further explore the protective role of Spd in tall fescue leaves against heat stress. We observed that exogenous Spd made tremendous contributions toward enhancing the expression levels of *psbA* and *psbB*, but it did not affect on *psbC* under the normal conditions (**Figure 9**). Heat stress suppressed the expression of the three genes compared to normal temperature. However, Spd application profoundly promoted the *psbA* and *psbB* expressions while did not affect on *psbC*.

## Discussion

Tall fescue, as a typical cool-season turf and forage grass, is limited by heat stress. Therefore, it is vital to improving the thermotolerance for its efficient use in turf industry. It has been reported that Spd plays a crucial role of against abiotic stresses, such as salt, drought, heat, and salinity–alkalinity stresses in tomato, cucumber, rice (Farooq et al., 2009; Tian et al., 2012; Hu L. et al., 2016). In the present study, we would explore the alleviative effect of Spd to heat stress in tall fescue.

Cell membrane breakage is one of the major damages caused by heat stress which leads to cell permeability and EL (Liu and Huang, 2000). Therefore, the EL could usually be used as an indicator to reflect the damage of membrane in heat stress (Blum and Ebercon, 1981; Marcum, 1998). It was reported that exogenous application of Spd could induce endogenous PAs generating (Li et al., 2016). And PAs, in the state of polycation, could attach to the phosphate groups with anions which results in stability of the membrane and intracellular material outflow (Galston and Sawhney, 1990). Additionally, PAs are ideally suitable for physiological channel modulator to block fast vacuolar channel activity and K^+^/Na^+^ homeostasis partially at a physiological pH (Alcázar et al., 2010). Moreover, some studies previously indicated that PAs can also non-covalently bind to some low molecules including proteins in the plasma membrane, antibiotics, phenolic acids, and hydroxycinnamic acid (Feuerstein and Marton, 1989; Martin-Tanguy, 2001). In the present study (**Figure 2**), the value of EL soared after high-temperature treatment which suggested that tall fescue leaf cells experienced serious EL. However, the EL was then reduced after Spd treatment. Those results indicated that exogenous applied Spd could induce the PAs generating in tall fescue leaf cells. Besides, PAs may interact with negatively charged phosphate groups and low molecules mentioned above. As a result, the treatment of Spd had a positive effect on balancing the pH and ionic exchange through plasma membrane. Therefore, exogenous Spd could decrease the EL of tall fescue leaf cells which under heat stress.

Under optimum conditions, the plants could balance the generating and scavenging ROS (Bowler et al., 1992) through a well-organized scavenging system (Tian et al., 2012). However, the ROS, such as H_2_O_2_ and O_2_^⋅-^, will largely accumulate when the plants suffer from various abiotic stress. These abiotic stresses could lead to plant metabolic inactivation, cell death, net photosynthesis rate and photosynthate reduction or even cause the loss of plant quality and serious yield reduction (Mallick and Mohn, 2000). Under heat stress, the ROS production always exceeds the defense capability of antioxidant resulting in macromolecules being damaged in living cells (Liu and Huang, 2000; Tian et al., 2012). The SOD-POD and APX line plays crucial role in scavenging ROS. The SOD could catalyze O_2_**^⋅-^** to H_2_O_2_ and oxygen (Sigaud-Kutner et al., 2002). The H_2_O_2_ can be further removed by POD and APX through dismutation or ascorbateglutathione cycle afterward (Mostofa et al., 2014; Liu et al., 2016). In the present study, the H_2_O_2_, O_2_**^⋅-^**, SOD, POD, and APX activities were measured to explore the effect of Spd in scavenging ROS and promoting antioxidant enzymes activities (**Figures 4, 5**). The result showed that exogenous Spd could significantly decrease the H_2_O_2_ and O_2_^⋅-^ contents which indicated the application of Spd alleviating the heat stress suffered by tall fescue leaves. On the other hand, antioxidant enzymes activities increased obviously regardless of whether the activities of these enzymes were inhibited or raised by high-temperature with the treatment of Spd. Shi et al. (2013) has reported that Spd could regulate nucleoside diphosphate kinase (NDPK) and three antioxidant enzymes (2-Cys POD, APX, Cu/Zn SOD) in bermudagrass. PAs increased level of NDPK_2_ protein which is directly related with activated activities of antioxidant enzymes (Shi and Chan, 2014). Namely, exogenous PAs treatment regulated the level of NDPK_2_ protein related with activating the activities of antioxidant enzymes to inhibit accumulations of O_2_^⋅-^ and H_2_O_2_ under stress conditions (Shi et al., 2013). In addition, exogenous Spd may alter the antioxidant enzymes isozymes zymogram expression, which could also enhance plant tolerance at high temperature (Tian et al., 2012). Some studies reported that PAs could also be as efficient antioxidants by their character of polycation under various environmental stresses (Groppa et al., 2001). From the results, we observed that exogenous Spd could have the positive effects on inhibiting ROS generation and promoting antioxidative enzymes activities. Therefore, we also deduce that Spd could enhance the tall fescue thermotolerance resulting from removing ROS by regulating the expression of relevant gene.

Photosynthesis, inhibited by cold, heat, drought, saline-alkaline, and nutritional deficiency, is one of the most processes sensitive to diverse abiotic stresses (Jiang et al., 2001; Bi et al., 2016; Hu Z. et al., 2016; Xiang et al., 2016). PSII, as an important membrane structure of the photosynthesis processes, is also vulnerable to high temperature (Chen et al., 2014; Bi et al., 2016). Chlorophyll fluorescence kinetics transients could provide abundant information of the original photochemical reaction of the mechanism of photosynthesis, mainly in PSII donor side, receptor side, and reaction centers (Murkowski, 2001; Chen et al., 2014). To figure out the adaption mechanism of high temperature in tall fescue leaves treated by Spd, chlorophyll fluorescence transients and JIP-test were investigated. As shown in **Table 2** and **Figure 6**, there was subtle difference between the treatments regardless of application of Spd under normal condition. However, heat stress aggravated the function of PSII as observed after altering the F_0_, F_J_, F_I_, F_M_, and M_0_. Exogenous Spd obviously alleviated the inhibition of heat stress through observing the values which mentioned above. The difference indicates that exogenous Spd is vital to tall fescue heat stress-resistance by protecting the PSII. This result accorded with Murkowski (2001) study that adequate concentration Spd could alleviate heat stress damage of tomato PS II. Then we evaluated the quantum yields and efficiencies by maximum quantum yield for primary photochemistry (φP_0_, TR_0_/ABS), efficiency with which an electron from QB is transferred to PSI acceptors (δR_0_, RE_0_/ET_0_), the quantum yield for reduction of end electron acceptors at the PSI acceptor side (φR_0_, RE_0_/ABS), and the probability that a PSII Chl molecule functions as RC (γRC) in PSII. The result indicated that heat stress notably decreased the efficiency of electron transportation and Chl molecule functions of PSII. Exogenous Spd remarkably improved the φP_0_, δR_0_, φR_0_, and γRC. Videlicet, Spd has a positive effect on the side of donor and acceptor of PSII under high temperature. Specific energy fluxes including TP_0_/RC and ET_0_/RC were analyzed to detect the functional properties of PSII (**Figures 7E,F**). The result determined that the plant could not balance between light absorption and utilization under heat treatment, and it had a negative effect on the RC. After application of Spd, the trapped excitation flux (leading to Q_A_ reduction) and electron transport flux (further than Q_A_^-^) per RC resumed to normal level. These indicate that Spd increased the active RC and alleviated the damage on RC under the heat stress. Performance index (PI) including PI_ABS_ and PI_total_, is the most sensitive parameters of the JIP-test which is used for assessing the photochemical activities of stressed plants (Fan et al., 2015). The PI integrates several parameters containing light energy absorption, excitation energy trapping, and conversion of excitation energy to electron transport (Fan et al., 2015). In the present study, we ascertained that Spd treatment has a positive effect on PI_total_ and PI_ABS_ which were much higher than heat stress alone (**Figure 8**). As analyzed above, we can conclude that exogenous Spd has protective effects on PS II which is a very heat-sensitive membrane structure. To a certain degree, the results in PSII are consistent with Chen et al. (2013) studies on NO alleviation of heat damage in tall fescue.

The chorophyll content of leaves displays crucial information concerning the physiological condition of the plants (Gitelson et al., 2003). The chlorophylls, Chl a and Chl b, are really important pigments in the process of photosynthesis which related to the transforming light energy to chemical energy (Gitelson et al., 2003). It is known that both Chl a and Chl b can absorb light energy, but only a handful of the excited state Chl a can transform light energy into electrical energy. Chl b is a pigment of antenna complexes in green algae (Green and Durnford, 1996). It also plays an important role in regulating the size of the photosynthetic antenna and maintaining the stability of light-harvesting complex associated with PSII (LHCII) in plants (Yamasato et al., 2005). The ratio of chlorophyll a to b (Chl a/b) is a vital value of LHCII to other components that contain chlorophyll (Leong and Anderson, 1984). Tanaka et al. (2001) reported that the overexpression of *CAO* (chlorophyllide an oxygenase) in *Arabidopsis thaliana*, a key enzyme in the process of synthesis of Chl b, could increase the expression of antenna by 20%, and LHCII and CP47 content also increased by 40%. However, heat stress may induce a decline in chlorophyll level and the ratio of Chl a/Chl b in tall fescue leaves (Bi et al., 2016). In the present study, the content of Chl a, Chl b, and total chlorophyll content decreased under the high-temperature treatment. The impact on degrading chlorophyll by heat stress was improved after application Spd. As well, the ratio of Chl a to Chl b was higher after Spd treatment under heat stress. The result implies that exogenous Spd may act as a regulator to prevent chlorophyll from disintegrating and thus further protecting the photosynthetic antenna and structure of PSII to improve the heat tolerance and photosynthetic efficiency of tall fescue. Heat stress may induce that chlorophyll decreasing by generating ROS. We deduced that the Spd could improve the chlorophyll content via scavenging ROS, directly or indirectly. Previous study also indicated that low chlorophyll content also has disadvantages to photosynthesis and signaling (Brestic et al., 2016). That is according with our results of JIP-test. High temperature has a negative effect on chlorophyll and further influence on the electron transforming resulting to the function of PS II been damaged.

In higher plants, CP43 (*psbC* encoded protein) and CP47 (*psbB* encoded protein) are the core antenna protein complexes with the composition of chlorophyll a located in the RC of PSII (Bricker, 1990; Barber, 2003). They can transfer the excitation energy (captured by the peripheral antenna chlorophyll a/b protein complex, LHCII) to RC and also be involved in water splitting and maintain PSII core complex structure (Bassi et al., 1987; Bricker, 1990). D1 protein is the most essential subunit which can bind to cofactors, protect the structure of PS II RC, and have a close connection with the separation and transmission of the original chemical reaction (Kruse et al., 1997). Under high temperature, CP43 will uncouple the light harvesting antenna from the RC (Yoshioka et al., 2006). The damaged D1 protein will also splits after CP43 is released from RC, then the active RCs decrease leads to inefficient energy utilization as a result of a reduction in CP43 and CP47 (Vani et al., 2001). The data in **Figures 7–9** agree with these reports. However, the application of Spd promoted the transcription of CP47 and improves the behavior of RC to some extent. Thus, Spd could partially alleviate and heat damage for RC. D1 protein encoded by *psbA* is the most fundamental structure of PSII and a variety of cofactors related to the original charge separation and electron transfer are combined in an orderly in this structure (Bredenkamp and Baker, 1994). It was reported that a D1 protein was dramatically damaged when exposed to singlet oxygen produced from the water-oxidation reaction (Takahashi et al., 2004). Therefore, the protection and recovery of D1 proteins damaged by heat stress which are crucial for the RC of PSII. As observed in this study, the expression of *psbA* increased significantly in the presence of Spd, which could be a contributory toward the stability of PSII under heat stress. Polyamines can stabilize the structure of nucleic acids by its cation combined with the negative charge on phosphate groups of nucleic acid. It has been reported that exogenous PAs can protect RNA from the degradation of ribonuclease (Goyns, 1982). PAs may also as a mediator or secondary messenger to activate gene network with a potential to protect plants from biotic and abiotic stresses (Paschalidis and Roubelakis-Angelakis, 2005; Cona et al., 2006). It has also been reported that a number of genes were activated by PAs. These genes mainly about the transcription, translation, signal transduction, stress protein biosynthesis (Cheng et al., 2012). Exogenous Spd may regulate gene expression as discussed above. However, the detail of the mechanism is still unclear.

## Conclusion

Exogenous PAs has been regarded as an efficient method to alleviate plants heat stress. In this research, we found that exogenous Spd could improve the heat tolerance of tall fescue by protecting all kinds of membranes structures from ROS. As well, it has positive effects on increasing antioxidant enzymes activities and stabilizing the structure of nucleic acids. Besides, the heat damage to PS II of tall fescue is also been alleviated.

## Author Contributions

JF and YX designed the experiments. LZ performed the experiments, and wrote the manuscript. GW assisted with doing the experiments. TH analyzed the data. JF and EA helped to draft the manuscript and revised manuscript. All authors read and approved the final manuscript.

## Conflict of Interest Statement

The authors declare that the research was conducted in the absence of any commercial or financial relationships that could be construed as a potential conflict of interest.
